# GWAS of Tau‐Neurodegeneration Mismatch Identifies New Risk Loci for Susceptibility to Tau

**DOI:** 10.1002/cns.71125

**Published:** 2026-09-01

**Authors:** Fardin Nabizadeh, Hanieh Mohamadi, for the Alzheimer's Disease Neuroimaging Initiative (ADNI), Michael W. Weiner, Paul Aisen, Ronald Petersen, Clifford R. Jack, William Jagust, John Q. Trojanowski, Arthur W. Toga, Laurel Beckett, Robert C. Green, Andrew J. Saykin, John Morris, Leslie M. Shaw, Zaven Khachaturian, Greg Sorensen, Lew Kuller, Marcus Raichle, Steven Paul, Peter Davies, Howard Fillit, Franz Hefti, David Holtzman, Marek M. Mesulam, William Potter, Peter Snyder, Adam Schwartz, Tom Montine, Ronald G. Thomas, Michael Donohue, Sarah Walter, Devon Gessert, Tamie Sather, Gus Jiminez, Danielle Harvey, Matthew Bernstein, Paul Thompson, Norbert Schuff, Bret Borowski, Jeff Gunter, Matt Senjem, Prashanthi Vemuri, David Jones, Kejal Kantarci, Chad Ward, Robert A. Koeppe, Norm Foster, Eric M. Reiman, Kewei Chen, Chet Mathis, Susan Landau, Nigel J. Cairns, Erin Householder, Lisa Taylor‐Reinwald, Virginia Lee, Magdalena Korecka, Michal Figurski, Karen Crawford, Scott Neu, Tatiana M. Foroud, Steven G. Potkin, Li Shen, Kelley Faber, Sungeun Kim, Kwangsik Nho, Leon Thal, Neil Buckholtz, Marylyn Albert, Richard Frank, John Hsiao, Jeffrey Kaye, Joseph Quinn, Betty Lind, Raina Carter, Sara Dolen, Lon S. Schneider, Sonia Pawluczyk, Mauricio Beccera, Liberty Teodoro, Bryan M. Spann, James Brewer, Helen Vanderswag, Adam Fleisher, Judith L. Heidebrink, Joanne L. Lord, Sara S. Mason, Colleen S. Albers, David Knopman, Kris Johnson, Rachelle S. Doody, Javier Villanueva‐Meyer, Munir Chowdhury, Susan Rountree, Mimi Dang, Yaakov Stern, Lawrence S. Honig, Karen L. Bell, Beau Ances, Maria Carroll, Sue Leon, Mark A. Mintun, Stacy Schneider, Angela Oliver, Daniel Marson, Randall Griffith, David Clark, David Geldmacher, John Brockington, Erik Roberson, Hillel Grossman, Effie Mitsis, Leyla de Toledo‐Morrell, Raj C. Shah, Ranjan Duara, Daniel Varon, Maria T. Greig, Peggy Roberts, Chiadi Onyike, Daniel D'Agostino, Stephanie Kielb, James E. Galvin, Brittany Cerbone, Christina A. Michel, Henry Rusinek, Mony J. de Leon, Lidia Glodzik, Susan De Santi, PMurali Doraiswamy, Jeffrey R. Petrella, Terence Z. Wong, Steven E. Arnold, Jason H. Karlawish, David Wolk, Charles D. Smith, Greg Jicha, Peter Hardy, Partha Sinha, Elizabeth Oates, Gary Conrad, Oscar L. Lopez, MaryAnn Oakley, Donna M. Simpson, Anton P. Porsteinsson, Bonnie S. Goldstein, Kim Martin, Kelly M. Makino, MSaleem Ismail, Connie Brand, Ruth A. Mulnard, Gaby Thai, Catherine McAdams‐Ortiz, Kyle Womack, Dana Mathews, Mary Quiceno, Ramon Diaz‐Arrastia, Richard King, Myron Weiner, Kristen Martin‐Cook, Michael DeVous, Allan I. Levey, James J. Lah, Janet S. Cellar, Jeffrey M. Burns, Heather S. Anderson, Russell H. Swerdlow, Liana Apostolova, Kathleen Tingus, Ellen Woo, Daniel H. S. Silverman, Po H. Lu, George Bartzokis, Neill R. Graff‐Radford, Francine Parfitt, Tracy Kendall, Heather Johnson, Martin R. Farlow, Ann Marie Hake, Brandy R. Matthews, Scott Herring, Cynthia Hunt, Christopher H. van Dyck, Richard E. Carson, Martha G. MacAvoy, Howard Chertkow, Howard Bergman, Chris Hosein, Ging‐Yuek Robin Hsiung, Howard Feldman, Benita Mudge, Michele Assaly, Charles Bernick, Donna Munic, Andrew Kertesz, John Rogers, Dick Trost, Diana Kerwin, Kristine Lipowski, Chuang‐Kuo Wu, Nancy Johnson, Carl Sadowsky, Walter Martinez, Teresa Villena, Raymond Scott Turner, Kathleen Johnson, Brigid Reynolds, Reisa A. Sperling, Keith A. Johnson, Gad Marshall, Meghan Frey, Barton Lane, Allyson Rosen, Jared Tinklenberg, Marwan N. Sabbagh, Christine M. Belden, Sandra A. Jacobson, Sherye A. Sirrel, Neil Kowall, Ronald Killiany, Andrew E. Budson, Alexander Norbash, Patricia Lynn Johnson, Joanne Allard, Alan Lerner, Paula Ogrocki, Leon Hudson, Evan Fletcher, Owen Carmichae, John Olichney, Charles DeCarli, Smita Kittur, Michael Borrie, T.‐Y. Lee, Rob Bartha, Sterling Johnson, Sanjay Asthana, Cynthia M. Carlsson, Adrian Preda, Dana Nguyen, Pierre Tariot, Stephanie Reeder, Vernice Bates, Horacio Capote, Michelle Rainka, Douglas W. Scharre, Maria Kataki, Anahita Adeli, Earl A. Zimmerman, Dzintra Celmins, Alice D. Brown, Godfrey D. Pearlson, Karen Blank, Karen Anderson, Robert B. Santulli, Tamar J. Kitzmiller, Eben S. Schwartz, Kaycee M. Sink, Jeff D. Williamson, Pradeep Garg, Franklin Watkins, Brian R. Ott, Henry Querfurth, Geoffrey Tremont, Stephen Salloway, Paul Malloy, Stephen Correia, Howard J. Rosen, Bruce L. Miller, Jacobo Mintzer, Kenneth Spicer, David Bachman, Stephen Pasternak, Irina Rachinsky, Dick Drost, Nunzio Pomara, Raymundo Hernando, Antero Sarrael, Susan K. Schultz, Laura L. Boles Ponto, Hyungsub Shim, Karen Elizabeth Smith, Norman Relkin, Gloria Chaing, Lisa Raudin, Amanda Smith, Kristin Fargher, Balebail Ashok Raj, Thomas Neylan, Jordan Grafman, Melissa Davis, Rosemary Morrison, Jacqueline Hayes, Shannon Finley, Karl Friedl, Debra Fleischman, Konstantinos Arfanakis, Olga James, Dino Massoglia, JJay Fruehling, Sandra Harding, Elaine R. Peskind, Eric C. Petrie, Gail Li, Jerome A. Yesavage, Joy L. Taylor, Ansgar J. Furst

**Affiliations:** ^1^ School of Medicine Iran University of Medical Sciences Tehran Iran

**Keywords:** AKAP9, Alzheimer's disease, GWAS, neurodegeneration, STXBP6, tau

## Abstract

**Background:**

In Alzheimer's disease (AD), neurodegeneration is primarily attributed to the accumulation of tau neurofibrillary tangles. However, the distribution patterns of both tau pathology and neurodegeneration vary across different brain regions and among individuals. Moreover, multiple factors may influence the relationship between tau burden and neurodegenerative processes. Identifying the genetic architecture associated with deviation in the tau‐neurodegeneration relationship can provide deeper mechanistic insights and guide the development of precision medicine strategies.

**Methods:**

Here, I perform a genome‐wide association study (GWAS) of cortical tau and thickness quantified by positron emission tomography (PET) and magnetic resonance imaging (MRI) in 794 participants from two cohorts of Alzheimer's disease Neuroimaging Initiative (ADNI) and A4.

**Results:**

A GWAS was identified between the Tau/Neurodegeneration residual and two novel loci on chromosomes 7 and 14, with two SNPs (rs9323573 and rs9784993) exceeding the genome‐wide significance threshold (*p* ≤ 5 × 10^−8^). SNP rs9323573 is located in STXBP6 on chromosome 14, while rs9784993 is in AKAP9 on chromosome 7, both of which were directly genotyped. The minor allele G of both SNPs (rs9323573, MAF = 0.221, *p* = 2.60 × 10^−8^; rs9784993, MAF = 0.197, *p* = 4.92 × 10^−8^) was associated with lower Tau/Neurodegeneration residuals, indicating higher‐than‐expected neurodegeneration given tau levels.

**Conclusion:**

GWAS of tau‐related neurodegeneration identified two novel genetic variants in the loci AKAP9 and STXBP6 leading to higher than expected regional neurodegeneration given the tau level. Identifying genetic factors involved in tau‐neurodegeneration mismatch may improve our understanding regarding the potential mechanistic downstream leading to susceptibility or resilience to tau pathology.

## Graphical abstract

This GWAS of tau/neurodegeneration mismatch in 794 ADNI and A4 participants identified two genome‐wide significant loci, STXBP6 and AKAP9. The risk alleles were associated with greater‐than‐expected neurodegeneration for a given tau burden, suggesting these genes may influence susceptibility to tau‐related neuronal injury.

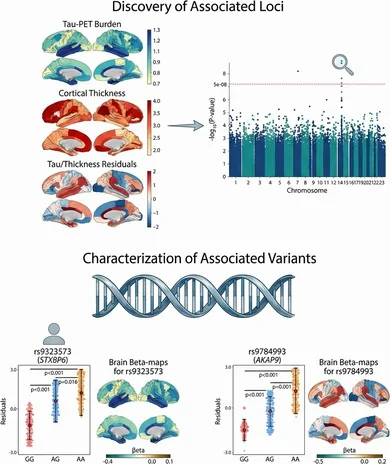

## 1 Introduction

Alzheimer's disease (AD) leads to cognitive impairment, characterized by considerable heterogeneity among patients in terms of clinical manifestations, as well as in the severity and distribution of pathological burden [1, 2]. In particular, clinicopathologic heterogeneity provides the opportunity to investigate AD biology and refine our understanding of disease progression, management, and treatment via biomarker‐based studies. The neuropathologic hallmarks of AD include the widespread accumulation of amyloid‐beta (Aβ) plaques within the neocortex and a hierarchically structured distribution of neurofibrillary tangles, predominantly composed of tau aggregates, in limbic and cortical association regions [3, 4, 5]. Specialized radiotracers are used in vivo for positron emission tomography (PET) imaging to target amyloid and tau aggregates, with examples including 18F‐Flortaucipir for detecting tau tangles [6, 7]. Neurodegeneration can be assessed through neuronal hypometabolism using 18F‐fluorodeoxyglucose (18F‐FDG) PET or through structural atrophy assessed via magnetic resonance imaging (MRI) [1, 8].

It is generally believed that neurodegeneration is largely driven by neurofibrillary tangles (NFTs) which are supported by postmortem and PET imaging demonstrating that tau exhibits a stronger association with downstream neurodegeneration compared to Aβ plaques [9, 10, 11, 12, 13]. Also, downstream neurodegeneration in AD is explained via the local presence and distribution of tau pathology [14, 15, 16]. However, both tau and neurodegeneration exhibit variability in their distribution patterns across the brain and among individuals, and the relationship between tau and neurodegeneration does not represent a complete one‐to‐one correspondence [17, 18, 19]. Typically, there is an association between neurofibrillary tangle deposition and neuronal loss. However, deviations from this relationship can occur, such as cases where patients exhibit lower‐than‐expected neurodegeneration relative to their tau level or higher‐than‐expected neurodegeneration relative to their tau level. Quantifying the degree of tau‐neurodegeneration mismatch may provide insight into neuronal metabolic resilience or vulnerability to tau, potentially associated with non‐AD pathophysiological mechanisms or genetic factors [20]. Previous studies have supported the role of the APOE (apolipoprotein E) ε4 allele (APOE4) as a driver of higher‐than‐expected neurodegeneration given the tau pathology load or in other words susceptibility to tau pathology [19, 20, 21].

Genetic factors conferring susceptibility to or protection from AD are important for identifying biological pathways for drug development and personalized medicine [22]. Consequently, elucidating these genetic contributors can yield critical insights that inform novel therapeutic strategies and guide the advancement of individualized treatment approaches. Extensive genome‐wide association studies (GWAS) employing case–control methodologies have identified risk genes implicated in immune response, tau pathology, Aβ deposition, lipid metabolism, and additional biological pathways [23, 24]. The most significant genetic risk locus for AD is the APOE4 [25]. Beyond APOE4, GWAS have identified nearly thirty independent loci associated with disease risk [26, 27, 28]. From a pathophysiological standpoint, the risk genes discovered thus far predominantly influence AD through the modulation of immune system responses, endocytic pathways, cholesterol homeostasis, and APP catabolic processes [26, 27]. However, large case–control studies often face limitations due to the unknown neuropathological status of participants.

Genetic determinants are likely to modulate the extent and impact of tau pathology on neuronal integrity and brain function, thereby affecting the progression and severity of tau‐mediated neurodegenerative processes [19, 29, 30]. Endophenotype studies augment case–control approaches by evaluating genetic variants related to disease pathology [31, 32, 33]. A study examined the association between [18F]flortaucipir PET and BIN1, identifying a correlation between the BIN1 risk single nucleotide polymorphism (SNP; rs744373) and increased tau [34]. Another genome‐wide association study (GWAS) on tau PET endophenotypes discovered two genetic loci, PPP2R2B and IGF2BP3, although it was constrained by a modest sample size (*n* = 754) and the absence of a replication cohort [35, 36]. Despite the significant progress made in AD genetics, questions such as what the genetic factors related to tau‐induced neurodegeneration remain unanswered.

In this study, I conducted the GWAS of PET‐based cortical tau and cortical thickness, incorporating data from two independent cohorts. I investigated the association between the top SNP and deviation in the tau‐neurodegeneration relationship. My methodology includes gene‐set enrichment analysis, assessment of gene expression levels in human brain tissue and single‐nucleus RNA‐Seq data, mapping gene expression within the Allen Human Brain Atlas, and performing methylation and expression quantitative trait loci (eQTL) analyses.

## 2 Methods

### 2.1 Participants

Detailed descriptions of each cohort can be found in Tables S1 and S2. Participants were retrieved from the Alzheimer's Disease Neuroimaging Initiative (ADNI; http://adni.loni.usc.edu) and the Anti‐Amyloid Treatment in Asymptomatic Alzheimer's study (A4). Participants were included if they had (i) a tau PET scan and (ii) a T1‐weighted MRI acquired within 6 months of each other and complete data for age, sex, and principal components. Participants were excluded for missing key covariates, failed imaging QC, or major non‐AD neurologic conditions. When multiple scans were available, we selected the PET–MRI pair with the shortest inter‐scan interval.

### 2.2 Genotyping and Imputation

Genotyping of participants was conducted across multiple platforms. For each cohort, untyped single nucleotide polymorphisms (SNPs) were imputed using the Haplotype Reference Consortium (HRC) data as the reference panel [37]. Prior to imputation, standard quality control (QC) protocols were applied to both samples and SNPs [38]. Only non‐Hispanic individuals of European ancestry, as determined by multidimensional scaling analysis, were included [39]. The imputation and QC procedures followed established methodologies as previously described [40].

### 2.3 Statistical Analysis

#### 2.3.1 Definition of Tau/Neurodegeneration Mismatch

Normality of all continuous variables was assessed using the Shapiro–Wilk test. Tau–neurodegeneration mismatch was quantified as the residual from a region‐wise robust linear regression relating cortical thickness to tau‐PET SUVR. Specifically, for each of the 68 cortical ROIs, we fit: Thickness_ROI = β0 + β1·TauSUVR_ROI + ε, using a bi‐square weighting function to reduce the influence of outliers [20]. The mismatch residual was defined as observed thickness minus model‐predicted thickness for that ROI (in cortical thickness units). Thus, positive residuals indicate greater cortical thickness (less neurodegeneration) than expected given tau burden (relative resilience), whereas negative residuals indicate lower cortical thickness (more neurodegeneration) than expected given tau burden (relative vulnerability/susceptibility) [20].

#### 2.3.2 Genome‐Wide Association Analysis (GWAS)

Tau/Neurodegeneration mismatch, represented as residuals across all cortical regions, exhibited a normal distribution following a rank‐based inverse normal transformation. A genome‐wide association study (GWAS) on Tau/Neurodegeneration residuals was conducted using imputed genotypes within a linear regression framework, adjusting for age, sex, two principal components (PCs) to account for population stratification, APOE4 status, and diagnosis as covariates, implemented via PLINK. APOE4 status was included as a covariate to examine the additional influence of the identified AKAP9 and STXBP6 loci beyond the effect of APOE4 and to explore potential epistasis with APOE4.

#### 2.3.3 FUMA Analysis

To further investigate the genetic mechanisms underlying resilience to tau pathology, I conducted a FUMA analysis to functionally map and annotate genetic associations using GWAS summary results for Tau/Neurodegeneration residuals. FUMA is an online platform designed to streamline post‐GWAS analysis by integrating information from multiple resources, supporting functional annotation and gene prioritization [41]. FUMA comprises two key processes: SNP2GENE, which annotates SNPs for biological function and maps them to genes, and GENE2FUNC, which provides functional annotation of mapped genes within biological contexts.

Functional annotation was conducted using the SNP2GENE module in FUMA (v1.3.6) to prioritize, annotate, and interpret GWAS findings. Initially, significant independent SNPs from the GWAS summary statistics were identified based on a *p*‐value threshold (*p* < 5 × 10^−8^) and mutual independence (*r* ^2^ < 0.6) within a 250 kb window, using the 1000G Phase 3 EAS reference. Following this, lead SNPs—independent at an *r* ^2^ threshold of < 0.1—were isolated among the significant SNPs. SNPs in linkage disequilibrium (LD) with these independent SNPs (*r* ^2^ ≥ 0.6) within a 250 kb window were selected as candidate SNPs for further annotation.

FUMA annotates these candidate SNPs within genomic risk loci by assessing their functional impact on genes using ANNOVAR [42], CADD scores [43], regulatory function potentials (RegulomeDB scores) [44], and eQTL data across various tissues (GTEx v8) [45], as well as 3D chromatin interactions from 3D chromatin interaction mapping (Hi‐C) data across 21 tissue/cell types. The CADD score provides an estimate of SNP deleteriousness, with a score of 12.37 as a threshold for deleteriousness; scores ≥ 10 indicate variants within the top 10% for deleterious substitutions, while scores ≥ 20 fall within the top 1%. Gene mapping was conducted using positional mapping (based on ANNOVAR and a default SNP‐gene distance of 10 kb), eQTL mapping, and 3D chromatin interaction mapping. Only significant eQTLs (FDR < 0.05) and significant chromatin interactions (FDR < 1 × 10^−6^) were considered. GTEx Analysis Release V8 (dbGaP Accession phs000424.v8.p2) was further utilized for investigation.

A gene‐based analysis was performed using MAGMA v1.07 with default settings within the FUMA platform [45]. For FUMA, a total of 15,480 gene sets from MsigDB v7.0 were included, comprising 5497 curated gene sets and 9983 Gene Ontology (GO) terms. In the MAGMA analysis, SNPs were mapped to protein‐coding genes based on their genomic location, and the SNP *p*‐values were aggregated into a gene‐level test statistic using the SNP‐wise mean model. Bonferroni correction was applied across all gene sets analyzed. To assess tissue‐specific associations with the phenotype, FUMA conducted MAGMA gene‐property analyses, examining the relationships between tissue‐specific gene expression profiles and gene‐disease associations.

### 2.4 Gene⁓Neuroimaging Association

The influence of the identified SNPs on global tau, cortical thickness, and Tau/Neurodegeneration residuals was analyzed using an ANCOVA model, adjusting for age, sex, APOE ε4 carrier status, amyloid positivity, diagnosis, and the first two principal components (PCs) for population structure. Subsequently, linear regression was used to investigate the association between identified SNPs and regional tau, cortical thickness, Tau/Neurodegeneration residuals with adjustments made for age, sex, APOE ε4 carrier status, amyloid positivity, and the first two population PCs. Residuals were estimated across the full sample to capture the continuous tau–atrophy relationship over the study spectrum. This full‐sample model provides a single internal reference pattern spanning preclinical to clinical stages, consistent with our objective of characterizing tau–atrophy coupling across the AD continuum rather than within a single stage. Accordingly, the mismatch residual is interpreted as an individual's deviation from the study‐wide tau–cortical thickness relationship across cohorts. To account for stage‐related differences in mean levels of tau and neurodegeneration, diagnosis and amyloid positivity (where available) were included as covariates in downstream association models. Because tau signal is typically low in amyloid‐negative individuals, the biological interpretation of mismatch is most relevant to the Alzheimer's (A+) continuum; therefore, amyloid positivity and diagnosis were accounted for as covariates in downstream association models.

Data processing and statistical/bioinformatics analyses were performed using R version 4.1.2 (https://www.r‐project.org/), SMR (https://cnsgenomics.com/software/smr/), and FUMA (https://fuma.ctglab.nl/).

## 3 Results

### 3.1 Participants

**FIGURE 1:**
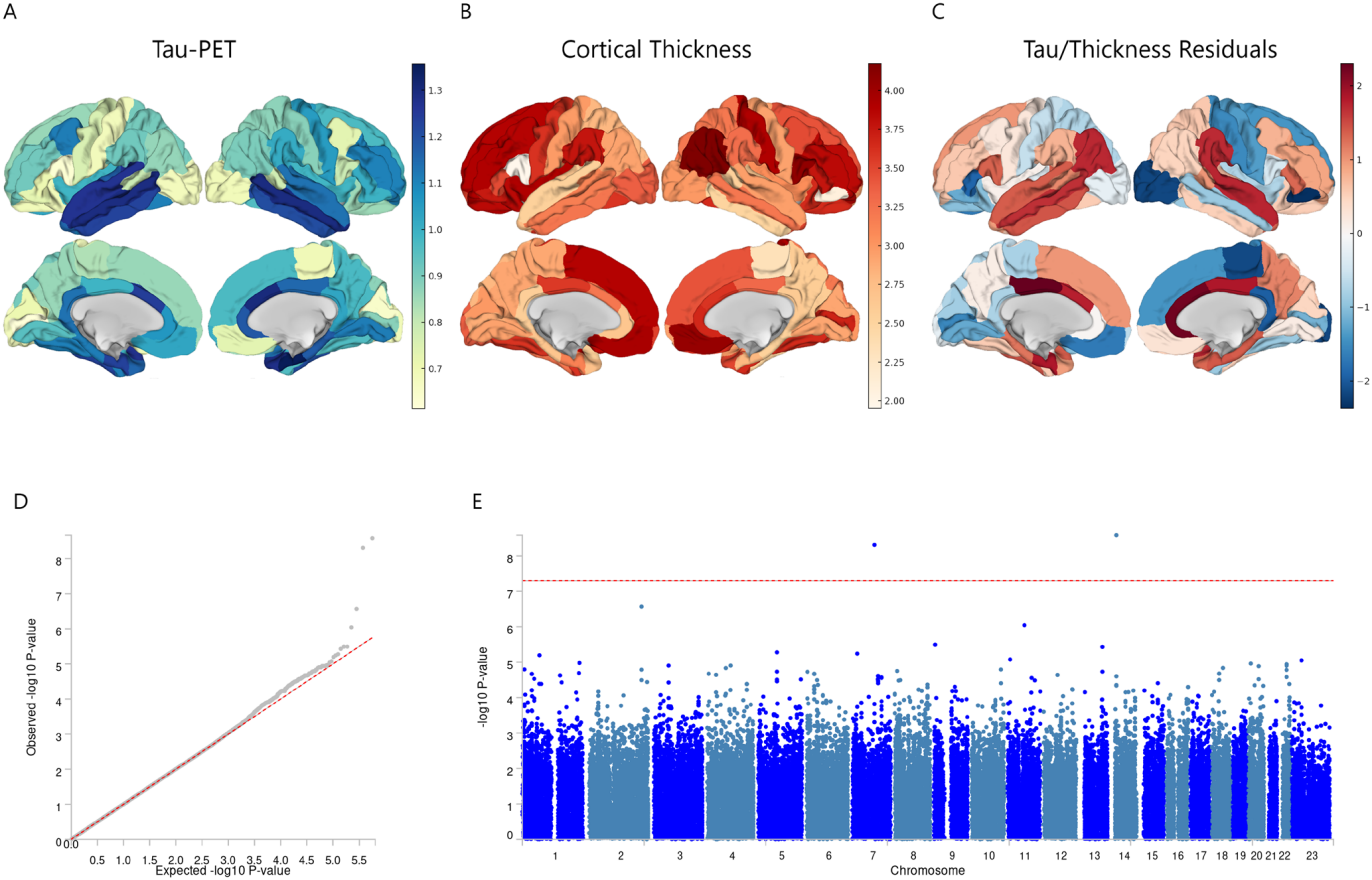
Results of GWAS analysis for Tau/Thickness residuals. (A) Surface rendering of tau‐PET SUVR from three included cohorts. (B) Surface rendering of cortical thicknesses from three included cohorts. (C) Surface rendering of tau/thickness residuals from three included cohorts. (D) Quantile‐quantile (QQ) and (E) Manhattan plots of genome‐wide association study (GWAS) results. The red line represents the significant threshold of two‐sided *p* < 5 × 10^−8^. Two single nucleotide polymorphisms (SNPs) on chromosomes 7 and 14 showed significant association with tau/thickness residuals in three included cohorts.

I conducted a meta‐analysis of GWAS on the association between tau‐PET SUVR and cortical thickness residual, incorporating data from two cohorts of ADNI and A4 (*n* = 794) (refer to Tables S1 and S2). All neuroimaging data were parcellated into 68 cortical regions. The highest level of tau‐PET SUVR was observed in temporal lobe regions among all subjects (Figure 1A). Also, the highest cortical thickness was in frontal lobe regions along with the involvement of some regions in the occipital lobe (Figure 1B). I calculated the deviation from the linear regression line of the association between tau‐PET SUVR and cortical thickness in each region across all participants as residual level. The higher residual level demonstrates lower neurodegeneration than expected given tau pathology while the lower level of residuals indicates the susceptibility to tau‐related neurodegeneration. The highest Tau/Neurodegeneration residuals were observed in the temporal and cingulate gyrus (Figure 1C).

### 3.2 Genome‐Wide Association Analysis (GWAS)

**FIGURE 2:**
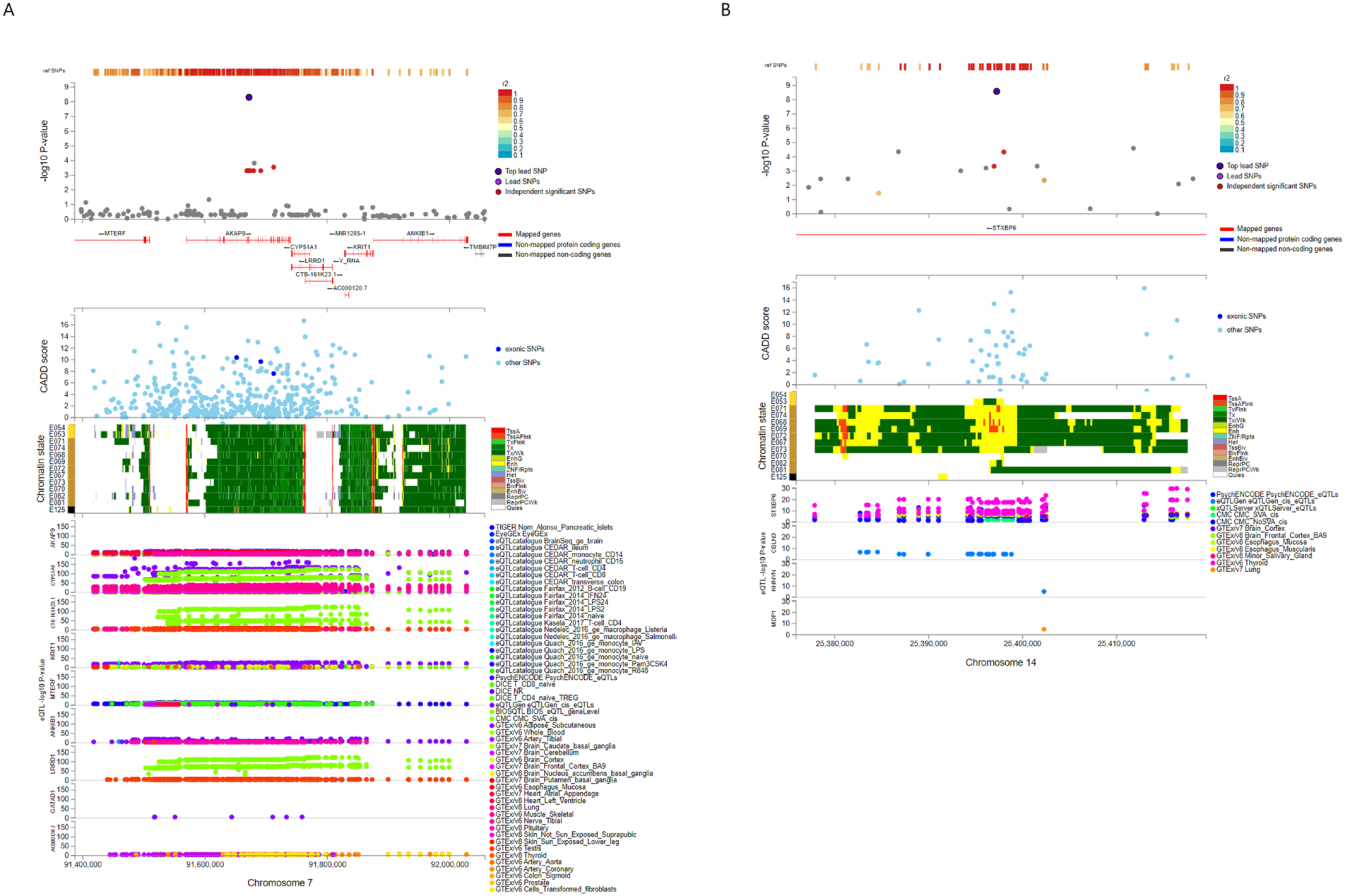
Mapping of the complex locus influencing tau‐related neurodegeneration (residuals) on chromosomes 7 and 14. (A) The regional plot for rs9784993 zoomed into a 450 kb region surrounding the AKAP9 gene. Zoomed regional plot of AKAP9 locus with CADD score, regulatory elements in the chromatin 15 state interaction plot for rs9784993, and eQTL *p*‐value. Each SNP is color‐coded based on the highest *r* ^2^. (B) The regional plot for rs9323573 zoomed into a 450 kb region surrounding the STXBP6 gene. Zoomed regional plot of STXBP6 locus with CADD score, regulatory elements in the chromatin interaction plot for rs9323573, and eQTL *p*‐value. Each SNP is color‐coded based on the highest *r* ^2^.

The results of the meta‐analyzed genome‐wide association study (GWAS) from two cohorts (*n* = 794) are presented as quantile‐quantile (Figure 1D) and Manhattan (Figure 1E) plots. No evidence of systematic *p*‐value inflation was observed. I identified a genome‐wide significant association between Tau/Neurodegeneration residual and two novel loci on chromosomes 7 and 14 (Figure 1E), with two single nucleotide polymorphisms (SNPs) surpassing the genome‐wide significance threshold (*p*‐value ≤ 5 × 10^−8^). The two lead SNPs, rs9323573 on chromosome 14 and rs9784993 on chromosome 7, were directly genotyped. SNP rs9323573 is located on chromosome 14 on STXBP6 (Figure 2B), and rs9784993 on chromosome 7 on AKAP9 (Figure 2A). The minor allele G of rs9323573 (minor allele frequency [MAF] = 0.221) was associated with lower Tau/Neurodegeneration residual (higher neurodegeneration than expected given tau) (*p* = 2.60 × 10^−8^). Furthermore, minor allele G of rs9784993 (minor allele frequency [MAF] = 0.197) showed the same association direction with lower Tau/Neurodegeneration residual (higher neurodegeneration than expected given tau) (*p* = 4.92 × 10^−8^) (Table S3). The two genomic loci of susceptibility to tau pathology‐related neurodegeneration were different in size, number of SNPs in high LD, and the number of genes physically located in the loci (Figures S1 and S2).

### 3.3 Functional Annotation

**FIGURE 3:**
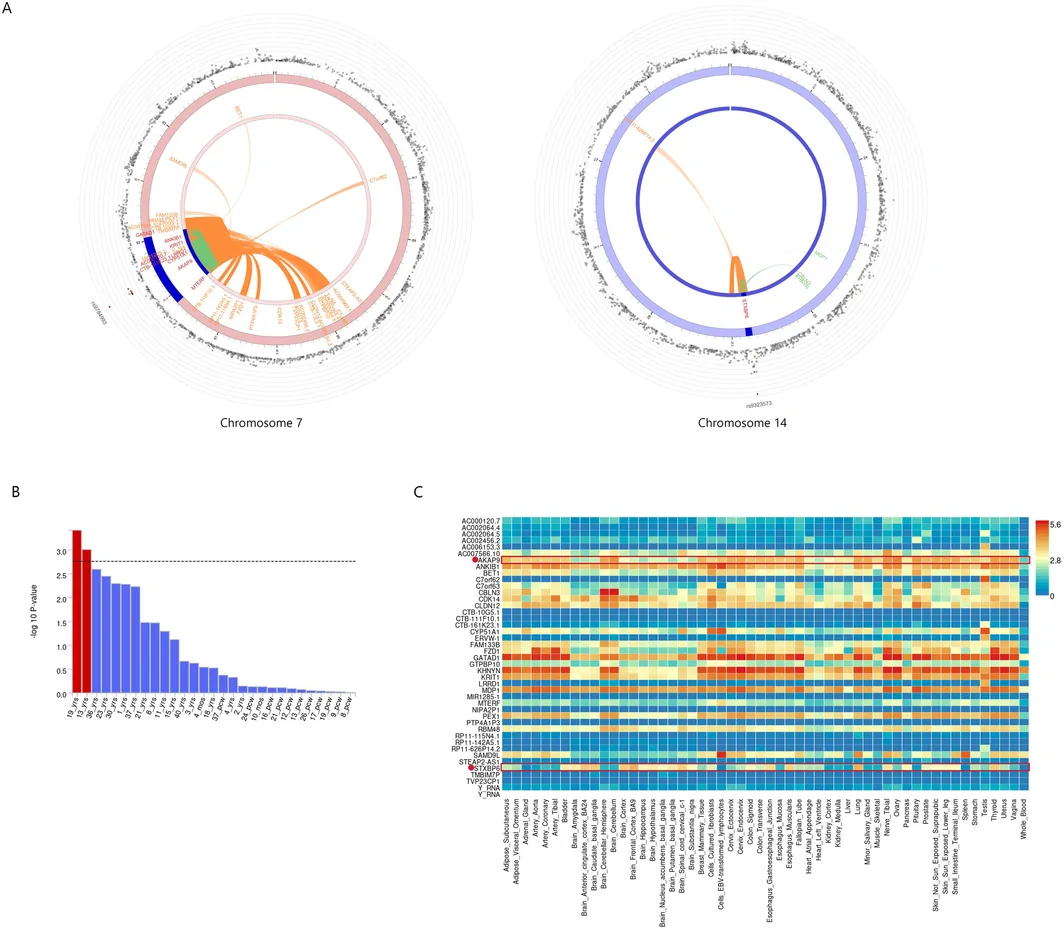
Tissue expression analysis. (A) 3D chromatin interaction (Hi‐C) and eQTL analysis based on GWAS of tau‐related neurodegeneration (residuals). (B) MAGMA tissue expression analysis using the BrainSpan 29 different ages of brain samples. (C) Tissue‐specific expression heatmap of AKAP9 and STXBP6 in 53 specific tissue types.

To examine the tissue specificity of the susceptibility to tau pathology‐related neurodegeneration, I conducted an analysis of tissue‐specific gene expression using MAGMA integrated within the FUMA platform. This approach enabled us to assess the associations between tissue‐specific gene expression patterns and genes linked to susceptibility to tau pathology‐related neurodegeneration across different ages of brain samples from the BrainSpan 29. The tau‐related neurodegeneration was significantly associated with genes expressed in the brain ages of 19 and 13 years (Figure 3B).

Hi‐C analysis revealed significant chromatin interactions (*r* ^2^ > 0.8) between SNPs associated with susceptibility to tau pathology‐related neurodegeneration (residuals) within the STXBP6 and AKAP9 region and multiple additional genes located on chromosomes 7 and 14 (Figure 3A). Among the genes identified through Hi‐C, 40 exhibit expression in 53 specific tissue types (Figure 3C) (Figure S3). The spatial distribution of AKAP9 and STXBP6 genes in the Allen brain atlas is depicted in Figures S4 and S5.

### 3.4 Association of Gene Risk Factors on Tau, Neurodegeneration, and Tau‐Related Neurodegeneration

**FIGURE 4:**
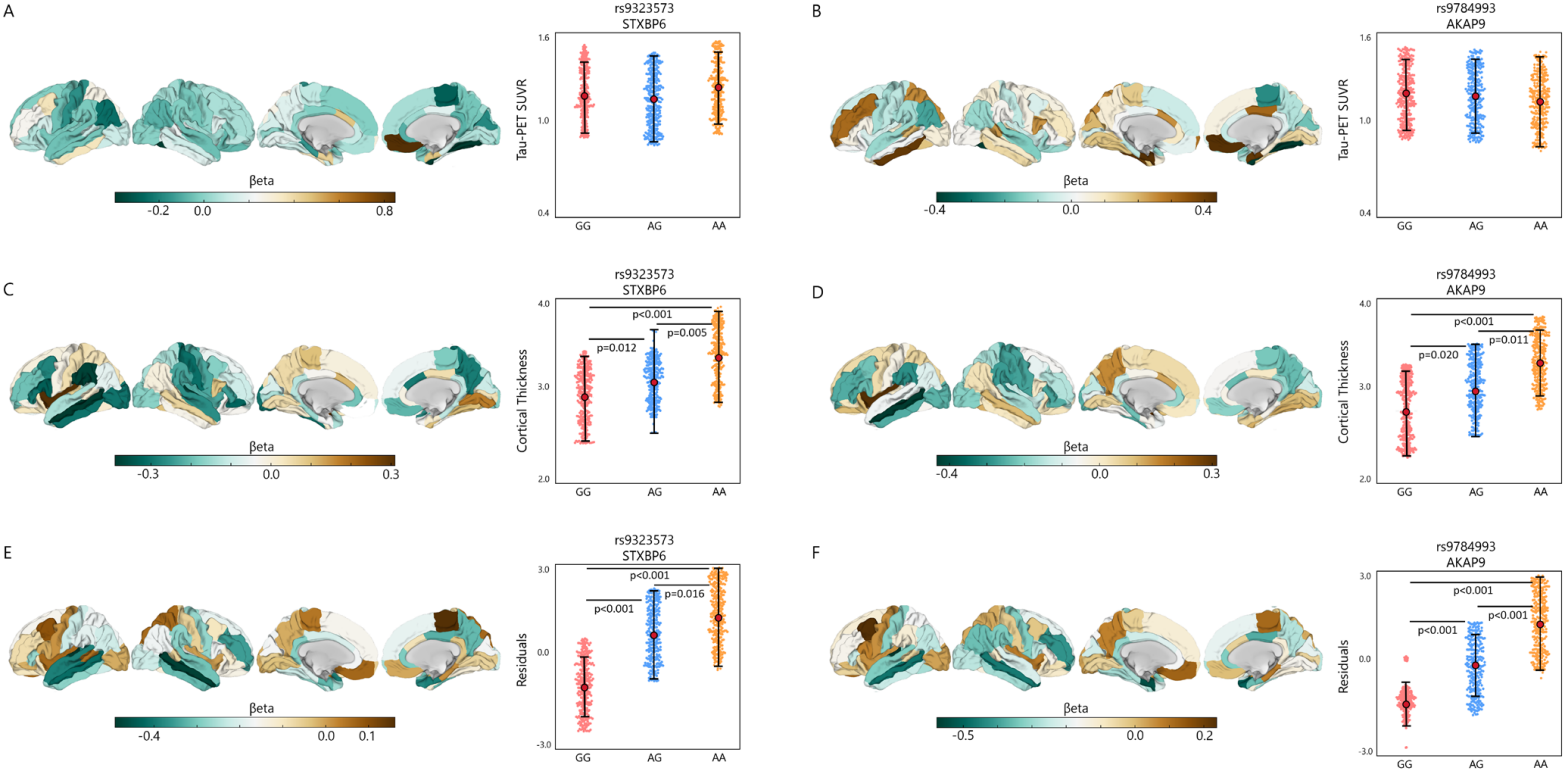
Association of significant SNPs with neuroimaging findings. (A) Surface rendering of regional association between tau‐PET SUVR and rs9323573. There was no significant association between overall tau‐PET and rs9323573. (B) Surface rendering of regional association between tau‐PET SUVR and rs9784993. There was no significant association between overall tau‐PET and rs9784993. (C) Surface rendering of regional association between cortical thickness and rs9323573. The allele G of rs9323573 was associated with lower cortical thickness. (D) Surface rendering of regional association between cortical thickness and rs9784993. The allele G of rs9784993 was associated with lower cortical thickness. (E) Surface rendering of regional association between tau/thickness residuals and rs9323573. The allele G of rs9323573 was associated with lower tau/thickness residuals (lower than expected neurodegeneration). (F) Surface rendering of regional association between tau/thickness residuals and rs9784993. The allele G of rs9784993 was associated with lower tau/thickness residuals (lower than expected neurodegeneration).

Following my mapping of genetic drivers of tau‐related neurodegeneration (residuals), I mapped the association between identified genetic risk factors and tau, neurodegeneration, and tau‐related neurodegeneration both globally and regionally. There was no difference in global tau‐PET SUVR value among allele G carriers and non‐carriers of rs9323573 (Figure 4A) and rs9784993 (Figure 4B). To determine whether genetic contributors of susceptibility to tau pathology‐related neurodegeneration were most strongly linked with regional tau, I applied linear regression models across 68 distinct brain regions. Higher tau in the right orbitofrontal and lower tau in the right fusiform was significantly associated with the rs9323573 genotype. Also, the rs9784993 genotype was significantly associated with lower tau‐PET SUVR in the right fusiform and higher tau‐PET in the left entorhinal.

The allele G carriers of both rs9323573 and rs9784993 had lower global cortical thickness compared to non‐carriers (Figure 4C,D). Regional analysis revealed lower cortical thickness in the left middle temporal and higher cortical thickness in the left insula was significantly associated with both rs9323573 and rs9784993 genotypes.

Further analysis showed that the allele G carriers of both rs9323573 and rs9784993 had lower Tau/Neurodegeneration residual (higher neurodegeneration than expected given tau) compared to non‐carriers (Figure 4E,F). At the regional level, it was determined that lower residuals in middle temporal regions were associated with the rs9323573 genotype. Also, the rs9784993 genotype was associated with lower residuals in the left transverse temporal and entorhinal regions.

## 4 Discussion

I performed a GWAS on the deviation from the one‐to‐one association between cortical tau and thickness in order to identify the genetic contributors of vulnerability or susceptibility to tau‐related neurodegeneration. I replicated two novel SNPs at the STXBP6 and AKAP9 locus with allele G associated with higher neurodegeneration than expected given tau. Regional exploratory analyses revealed that the rs9323573 genotype might affect tau‐related neurodegeneration in middle temporal regions while the rs9784993 genotype was associated with higher neurodegeneration than expected in the transverse temporal and entorhinal regions. Recent large‐scale GWAS studies on AD have indicated that the two SNPs do not exhibit a statistically significant association with AD, as demonstrated by varying directions of association across different studies (*p*‐value > 0.05) [23, 26, 46]. This absence of significant correlation may be attributable to heterogeneity in case–control selection based on clinical diagnosis, aligning with findings from quantitative endophenotype analysis that suggest a selective association. This work extends our previous study on Tau/Neurodegeneration mismatch, in which we characterized regional vulnerability and resilience using PET and MRI data [20].

The locus identified in this analysis contains two protein‐coding genes, AKAP9 and STXBP6, which exhibit high expression levels in the temporal regions of the brain (Figures S4 and S5) which are commonly affected by tau deposition and neurodegeneration in the course of AD. This spatial overlap suggests a potential relationship between gene expression patterns and tau‐associated neurodegenerative processes. Whole‐exome sequencing in a cohort of African American individuals with AD previously identified variants in AKAP9 [47]. AKAP9 encodes a scaffold protein that interacts with various kinase‐related molecules, including the regulatory subunit of protein kinase A (PPKAR2A) [48], CDK5RAP2 [49], and GSK3B [50]. This gene is broadly expressed in both the central nervous system and peripheral tissues. Additionally, AKAP9 has been implicated in long QT syndrome [51]. A study demonstrated that lymphoblastoid cell lines derived from individuals carrying the AKAP9 variants exhibited significant changes in the phosphorylation of recombinant Tau protein following virus‐mediated transient Tau expression [52]. These alterations in Tau phosphorylation occurred independent of AD status and APOE genotype. Another study demonstrated that the AKAP9 I2558M mutation, associated with AD, leads to a significant increase in phosphorylated Tau protein specifically at residues S396 and S404 in a differentiated cell line [53]. Furthermore, the mutation influenced the composition of Tau‐interacting proteins, enhancing interactions with proteins involved in translation, RNA localization, and oxidative activity. The AKAP9 mutation leads to reduced protein synthesis activity and increased oxidative stress in a tau‐dependent manner. However, a combination of my results and the lack of evidence regarding the association between AKAP9 and tau pathology level in non‐African populations might suggest that these genetic factors might be involved in susceptibility to tau in terms of neurodegeneration rather than the level of tau itself.

Interpretation of tau–neurodegeneration mismatch can vary by brain region. [18F]flortaucipir demonstrates its strongest disease‐relevant signal in canonical temporolimbic regions where tau pathology and neurodegeneration often co‐occur; outside these regions, apparent discordance should be interpreted cautiously. In the present study, the regional mismatch associations were concentrated in temporal areas (middle temporal, transverse temporal, entorhinal) rather than diffusely across cortex, which is broadly consistent with the expected spatial pattern of tau‐related neurodegeneration across the AD continuum. Importantly, mismatch may also reflect differences in temporal trajectories; for example, tau accumulation may precede measurable cortical thinning so a cross‐sectional residual can capture an earlier stage of neurodegeneration that is not yet detectable structurally rather than true biological dissociation.

STXBP6 was initially identified as a protein highly enriched in the brain, possessing a tomosyn‐ and VAMP‐like coiled‐coil‐forming domain. This domain enables STXBP6 to specifically bind to syntaxin‐1, thereby playing a regulatory role in SNARE complex formation [54]. Subsequent research identified STXBP6 as a negative regulator of exocytosis, acting by disrupting fusion pore formation [55]. Although STXBP6 plays a critical role in neuronal vesicle trafficking and has been implicated in neurological conditions such as developmental epileptic encephalopathy and autism spectrum disorders, no direct evidence links it to tau or neurodegeneration [56]. The potential connection to tau‐related neurodegeneration would likely stem from broader mechanisms affecting neuronal function rather than a direct interaction with tau proteins.

The relationship between tau and neurodegeneration AD exhibits variability due to the non‐specific nature of neurodegeneration. This variability is influenced by non‐AD co‐pathologies, age‐related changes, and resilience factors that modulate the extent of neurodegeneration [21]. Along with previously identified non‐AD copathologies such as TDP‐43 which might cause deviation in the one‐to‐one association between tau and neurodegeneration, APOE ε4 was found to be associated with higher than expected neurodegeneration given the regional tau level [19, 21]. This is the first GWAS study to investigate the potential genetic variants of tau‐related neurodegeneration in order to identify SNPs that are involved in the susceptibility or vulnerability to subsequent neurodegeneration of tau pathology. The suggested two lead SNPs may improve our understanding of the biology and prognostication of individuals with AD and offer valuable insights into the characterization of phenotypic heterogeneity within clinical populations, which could have significant implications for therapeutic trials, particularly in the context of resilience factors. Although the present study focuses on individuals within the AD continuum, tau‐related neurodegeneration is a shared feature across primary tauopathies, including progressive supranuclear palsy, corticobasal degeneration, and frontotemporal lobar degeneration with tau inclusions. Genetic factors that modulate susceptibility to neurodegeneration in the presence of tau pathology, such as the AKAP9 and STXBP6 loci identified here, may therefore have relevance beyond Alzheimer's disease, even if effect sizes, disease stages, and interacting copathologies differ across disorders. Future work in well‐characterized cohorts of non‐AD tauopathies will be important to determine whether the mechanisms suggested by our findings generalize across the broader spectrum of human tauopathy.

My study has some limitations. First, although linear regression was used to assess the tau‐neurodegeneration mismatch, the relationship between tau and neurodegeneration is likely to exhibit some degree of non‐linearity, even in cases of pure AD. Advanced non‐linear approaches, such as image‐to‐image translation, may provide a more accurate representation of tau‐neurodegeneration dynamics, including potential remote spatial dependencies between network hubs and their connected nodes. Second, since this study was primarily observational and included only cohorts of European ancestry, its generalizability to other populations remains limited. Multiethnic studies are essential, and validating these findings through large‐scale community studies or international collaborations is necessary to ensure broader applicability. While the two cohorts employed similar methodologies, minor variations may exist due to differences in Freesurfer versions or slight discrepancies in reference regions used for SUVR calculation. While sex differences are increasingly acknowledged as significant for precision medicine in AD, this study was not specifically designed or adequately powered to comprehensively investigate these effects. Future research with larger longitudinal cohorts is necessary to evaluate the presence and patterns of sex differences over time. Although two cohorts included in this study have longitudinal follow‐up data, the current analysis primarily focused on cross‐sectional associations. The cohort included a substantial proportion of cognitively unimpaired participants. While we adjusted for diagnosis (and amyloid positivity where applicable), we did not perform diagnosis‐stratified or A + ‐only GWAS due to reduced sample size and power; future larger datasets will be needed to confirm stage‐specific effects. A key limitation is the absence of replication in an independent cohort. Replication ideally requires a dataset with comparable tau PET and MRI measures and sufficient sample size for GWAS, which is not currently available to us within the scope of this revision. Our results should therefore be considered discovery findings that require confirmation in future external cohorts/consortia. A further limitation is the heterogeneity of the MCI group. Not all individuals diagnosed with MCI will convert to dementia or demonstrate progressive neurodegeneration, which may limit the applicability of the observed associations to neurodegenerative progression.

In summary, GWAS of tau‐related neurodegeneration identified two novel genetic variants in the loci AKAP9 and STXBP6 leading to higher‐than‐expected regional neurodegeneration given the tau level. Identifying genetic factors involved in tau‐neurodegeneration mismatch may improve our understanding regarding the potential mechanistic downstream leading to susceptibility or resilience to tau pathology. Furthermore, gaining insight into an individual's underlying genetic architecture may enhance the ability to assess susceptibility or resilience for tau‐related neurodegeneration and inform the selection of susceptible individuals for potential early intervention.

## Author Contributions

Data collecting, analysis, supervision, concept, and drafting were done by Fardin Nabizadeh.

## Funding

The authors have nothing to report.

## Ethics Statement

The protocol for the research project has been approved by a suitably constituted Ethics Committee of an institution, and it conforms to the provisions of the Declaration of Helsinki according to the ADNI study (adni.loni.usc.edu). The STROBE checklist was followed in this observational study.

## Consent

This manuscript has been approved for publication by all authors.

## Conflicts of Interest

The authors declare no conflicts of interest.

## Supporting information


**Table S1:** Demographic and clinical characteristics of ADNI.
**Table S2:** Demographic and clinical characteristics of A4.
**Figure S1:** Genetic risk loci identified by FUMA analysis using GWAS data on tau/neurodegeneration residual.
**Figure S2:** Functional consequences of SNPs on genes. The proportion of SNPs (all SNPs in LD of Ind. sig. SNPs) which have corresponding functional annotations assigned by ANNOVAR.
**Table S3:** Functional consequences of SNPs on genes.
**Figure S3:** Enrichment of input genes in Gene Sets.
**Figure S4:** Spatial distribution of AKAP9 gene in Allen brain atlas.
**Figure S5:** Spatial distribution of STXBP6 gene in Allen brain atlas.

## Data Availability

The data that support the findings of this study are available from the corresponding author upon reasonable request.

## Appendix A

Consortia and for the Alzheimer's Disease Neuroimaging Initiative: Michael W. Weiner, Paul Aisen, Ronald Petersen, Clifford R. Jack, William Jagust, John Q. Trojanowski, Arthur W. Toga, Laurel Beckett, Robert C. Green, Andrew J. Saykin, John Morris, Leslie M. Shaw, Zaven Khachaturian, Greg Sorensen, Lew Kuller, Marcus Raichle, Steven Paul, Peter Davies, Howard Fillit, Franz Hefti, David Holtzman, Marek M. Mesulam, William Potter, Peter Snyder, Adam Schwartz, Tom Montine, Ronald G. Thomas, Michael Donohue, Sarah Walter, Devon Gessert, Tamie Sather, Gus Jiminez, Danielle Harvey, Matthew Bernstein, Paul Thompson, Norbert Schuff, Bret Borowski, Jeff Gunter, Matt Senjem, Prashanthi Vemuri, David Jones, Kejal Kantarci, Chad Ward, Robert A. Koeppe, Norm Foster, Eric M. Reiman, Kewei Chen, Chet Mathis, Susan Landau, Nigel J. Cairns, Erin Householder, Lisa Taylor‐Reinwald, Virginia Lee, Magdalena Korecka, Michal Figurski, Karen Crawford, Scott Neu, Tatiana M. Foroud, Steven G. Potkin, Li Shen, Kelley Faber, Sungeun Kim, Kwangsik Nho, Leon Thal, Neil Buckholtz, Marylyn Albert, Richard Frank, John Hsiao, Jeffrey Kaye, Joseph Quinn, Betty Lind, Raina Carter, Sara Dolen, Lon S. Schneider, Sonia Pawluczyk, Mauricio Beccera, Liberty Teodoro, Bryan M. Spann, James Brewer, Helen Vanderswag, Adam Fleisher, Judith L. Heidebrink, Joanne L. Lord, Sara S. Mason, Colleen S. Albers, David Knopman, Kris Johnson, Rachelle S. Doody, Javier Villanueva‐Meyer, Munir Chowdhury, Susan Rountree, Mimi Dang, Yaakov Stern, Lawrence S. Honig, Karen L. Bell, Beau Ances, Maria Carroll, Sue Leon, Mark A. Mintun, Stacy Schneider, Angela Oliver, Daniel Marson, Randall Griffith, David Clark, David Geldmacher, John Brockington, Erik Roberson, Hillel Grossman, Effie Mitsis, Leyla de Toledo‐Morrell, Raj C. Shah, Ranjan Duara, Daniel Varon, Maria T. Greig, Peggy Roberts, Chiadi Onyike, Daniel D'Agostino, Stephanie Kielb, James E. Galvin, Brittany Cerbone, Christina A. Michel, Henry Rusinek, Mony J. de Leon, Lidia Glodzik, Susan De Santi, PMurali Doraiswamy, Jeffrey R. Petrella, Terence Z. Wong, Steven E. Arnold, Jason H. Karlawish, David Wolk, Charles D. Smith, Greg Jicha, Peter Hardy, Partha Sinha, Elizabeth Oates, Gary Conrad, Oscar L. Lopez, MaryAnn Oakley, Donna M. Simpson, Anton P. Porsteinsson, Bonnie S. Goldstein, Kim Martin, Kelly M. Makino, MSaleem Ismail, Connie Brand, Ruth A. Mulnard, Gaby Thai, Catherine McAdams‐Ortiz, Kyle Womack, Dana Mathews, Mary Quiceno, Ramon Diaz‐Arrastia, Richard King, Myron Weiner, Kristen Martin‐Cook, Michael DeVous, Allan I. Levey, James J. Lah, Janet S. Cellar, Jeffrey M. Burns, Heather S. Anderson, Russell H. Swerdlow, Liana Apostolova, Kathleen Tingus, Ellen Woo, Daniel H. S. Silverman, Po H. Lu, George Bartzokis, Neill R. Graff‐Radford, Francine Parfitt, Tracy Kendall, Heather Johnson, Martin R. Farlow, Ann Marie Hake, Brandy R. Matthews, Scott Herring, Cynthia Hunt, Christopher H. van Dyck, Richard E. Carson, Martha G. MacAvoy, Howard Chertkow, Howard Bergman, Chris Hosein, Ging‐Yuek Robin Hsiung, Howard Feldman, Benita Mudge, Michele Assaly, Charles Bernick, Donna Munic, Andrew Kertesz, John Rogers, Dick Trost, Diana Kerwin, Kristine Lipowski, Chuang‐Kuo Wu, Nancy Johnson, Carl Sadowsky, Walter Martinez, Teresa Villena, Raymond Scott Turner, Kathleen Johnson, Brigid Reynolds, Reisa A. Sperling, Keith A. Johnson, Gad Marshall, Meghan Frey, Barton Lane, Allyson Rosen, Jared Tinklenberg, Marwan N. Sabbagh, Christine M. Belden, Sandra A. Jacobson, Sherye A. Sirrel, Neil Kowall, Ronald Killiany, Andrew E. Budson, Alexander Norbash, Patricia Lynn Johnson, Joanne Allard, Alan Lerner, Paula Ogrocki, Leon Hudson, Evan Fletcher, Owen Carmichae, John Olichney, Charles DeCarli, Smita Kittur, Michael Borrie, T.‐Y. Lee, Rob Bartha, Sterling Johnson, Sanjay Asthana, Cynthia M. Carlsson, Adrian Preda, Dana Nguyen, Pierre Tariot, Stephanie Reeder, Vernice Bates, Horacio Capote, Michelle Rainka, Douglas W. Scharre, Maria Kataki, Anahita Adeli, Earl A. Zimmerman, Dzintra Celmins, Alice D. Brown, Godfrey D. Pearlson, Karen Blank, Karen Anderson, Robert B. Santulli, Tamar J. Kitzmiller, Eben S. Schwartz, Kaycee M. Sink, Jeff D. Williamson, Pradeep Garg, Franklin Watkins, Brian R. Ott, Henry Querfurth, Geoffrey Tremont, Stephen Salloway, Paul Malloy, Stephen Correia, Howard J. Rosen, Bruce L. Miller, Jacobo Mintzer, Kenneth Spicer, David Bachman, Stephen Pasternak, Irina Rachinsky, Dick Drost, Nunzio Pomara, Raymundo Hernando, Antero Sarrael, Susan K. Schultz, Laura L. Boles Ponto, Hyungsub Shim, Karen Elizabeth Smith, Norman Relkin, Gloria Chaing, Lisa Raudin, Amanda Smith, Kristin Fargher, Balebail Ashok Raj, Thomas Neylan, Jordan Grafman, Melissa Davis, Rosemary Morrison, Jacqueline Hayes, Shannon Finley, Karl Friedl, Debra Fleischman, Konstantinos Arfanakis, Olga James, Dino Massoglia, JJay Fruehling, Sandra Harding, Elaine R. Peskind, Eric C. Petrie, Gail Li, Jerome A. Yesavage, Joy L. Taylor & Ansgar J. Furst.
