# Supplementary material for: GWAS of Tau‐Neurodegeneration Mismatch Identifies New Risk Loci for Susceptibility to Tau
Source: CNS Neurosci Ther. 2026 Sep 1;32(9):e71125. doi: 10.1002/cns.71125 (PMC13532572; doi:10.1002/cns.71125)
Supplement: Supplementary file 1 [file CNS-32-e71125-s001.docx]

**Supplementary method**

**ADNI cohort**

The initial phase of the Alzheimer’s Disease Neuroimaging Initiative (ADNI-1), initiated in 2003, was designed to evaluate whether a combination of serial magnetic resonance imaging (MRI), positron emission tomography (PET), various biological markers, alongside clinical and neuropsychological assessments, could effectively monitor the progression of mild cognitive impairment (MCI) and early Alzheimer’s disease (AD). Following ADNI-1, subsequent phases (ADNI-GO, ADNI-2, and ADNI-3) have expanded the study to include longitudinal follow-ups with previous participants as well as the recruitment of new cohorts. Detailed descriptions of the ADNI project can be found on the official website (https://adni.loni.usc.edu). Publicly accessible data from the ADNI database includes demographic profiles, pre-processed tau PET imaging, APOE and whole-genome genotyping results, neuropsychological scores, and clinical assessments. The study adheres to established ethical guidelines, with all participant consent obtained in accordance with the Declaration of Helsinki, and has received approval from the relevant Institutional Review Boards. The analysis included a total of 501 participants from the ADNI cohort, comprising 289 cognitively normal older adults (CN), 164 individuals with MCI, and 48 with Alzheimer’s disease (AD). [18F]flortaucipir PET imaging was conducted following established protocols (http://adni.loni.usc.edu)5. Pre-processed PET scans for [18F]flortaucipir were acquired from the ADNI database and processed with Statistical Parametric Mapping (SPM12), incorporating normalization to Montreal Neurological Institute (MNI) space. Subsequently, [18F]flortaucipir standardized uptake value ratio (SUVR) images were produced by intensity normalization using the cerebellar crus as a reference region. The mean SUVR for [18F]flortaucipir was then extracted from a global cortical grey matter region, based on subject-specific parcellations of MPRAGE images generated through FreeSurfer version 6, employing regions defined in the Desikan-Killiany atlas6. Detailed procedures for cortical reconstruction and volumetric segmentation have been previously documented (1). Diagnostic group assignment (CN/MCI/AD) was based on the ADNI baseline clinical diagnosis as provided in the ADNI clinical database (i.e., diagnosis recorded at the baseline visit).

All images were visually inspected post-processing, and any errors in automated skull stripping or gray/white matter boundary detection were manually corrected before reanalysis. Cortical thickness measures were mapped to the white matter border or inflated cortical surface of each participant’s reconstructed brain for enhanced visualization, independent of cortical folding. For smoothing, a circularly symmetric Gaussian kernel with a full width at half maximum (FWHM) of either 20mm or 10mm was applied, and the data were averaged across participants using a high-dimensional spherical averaging technique to align cortical folding patterns. This method facilitated the precise anatomical matching of morphologically similar cortical regions across individuals while minimizing distortion, resulting in an averaged cortical thickness measure at baseline across reconstructed surfaces.

**A4 cohort**

The A4 study adheres to all pertinent ethical standards, with informed consent obtained from all participants in alignment with the Declaration of Helsinki, and has received approval from relevant Institutional Review Boards. The A4 trial is a 240-week pharmacological investigation assessing whether solanezumab, an anti-amyloid therapy, can slow cognitive decline and delay the onset of dementia in older adults exhibiting preclinical AD—specifically, cognitively normal individuals with amyloid positivity. Participants, recruited and evaluated at 67 clinical trial sites worldwide, were eligible if they demonstrated amyloid positivity via [18F]florbetapir PET imaging, maintained cognitive normality, and met all additional study criteria. Among these, a subgroup of 293 cognitively normal (CN) participants underwent tau PET imaging with [18F]flortaucipir. All A4 participants included here were cognitively normal at baseline per A4 eligibility criteria, and amyloid positivity was determined by [18F]florbetapir PET. These scans were obtained as 80-100 minute static images from the LONI data repository (https://ida.loni.usc.edu/home/projectPage.jsp?project=A4), co-registered with structural MRI images from the same visit, and normalized to Montreal Neurological Institute (MNI) space using segmentation parameters from structural MRI analysis with SPM12. Standardized uptake value ratio (SUVR) images were derived by normalizing to the cerebellar crus as a reference region, and mean [18F]flortaucipir SUVR was computed from a global cortical grey matter region defined by subject-specific parcellations of MPRAGE structural images, generated with FreeSurfer version 6. All images were visually inspected post-processing, and any errors in automated skull stripping or gray/white matter boundary detection were manually corrected before reanalysis. Cortical thickness measures were mapped to the white matter border or inflated cortical surface of each participant’s reconstructed brain for enhanced visualization, independent of cortical folding. For smoothing, a circularly symmetric Gaussian kernel with a full width at half maximum (FWHM) of either 20mm or 10mm was applied, and the data were averaged across participants using a high-dimensional spherical averaging technique to align cortical folding patterns. This method facilitated the precise anatomical matching of morphologically similar cortical regions across individuals while minimizing distortion, resulting in an averaged cortical thickness measure at baseline across reconstructed surfaces.

**Genotyping and Imputation**

Genotyping of participants was conducted using various Illumina platforms. Due to the use of different genotyping platforms across cohorts, imputation of un-genotyped single nucleotide polymorphisms (SNPs) was carried out independently for each cohort. This process utilized the MACH tool in conjunction with the Haplotype Reference Consortium (HRC) panel as a reference18. Prior to imputation, standard quality control protocols for samples and SNPs were applied as detailed previously19, including: (1) SNP exclusion criteria—SNP call rate below 95%, Hardy-Weinberg equilibrium p-value less than 1×10⁻⁶, and minor allele frequency (MAF) below 1%; (2) sample exclusion criteria—sex inconsistencies and sample call rate below 95%. To minimize potential confounding from population stratification, only non-Hispanic individuals of European ancestry clustering with HapMap CEU (Utah residents with Northern and Western European ancestry from the CEPH collection) or TSI (Toscani in Italia) populations were included, identified through multidimensional scaling (MDS) analysis (www.hapmap.org)20. The imputation and quality control processes were implemented as described previously21, with an r² threshold of 0.30 applied to accept imputed genotypes. Genotyping was conducted on Illumina arrays; ADNI participants were genotyped on the Illumina Human610-Quad (ADNI-1) and/or Illumina HumanOmniExpress (ADNI-GO/2) arrays, while A4 participants were genotyped on the Illumina Global Screening Array.

| Supplementary Table1. Demographic and clinical characteristics of ADNI | | | |
| --- | --- | --- | --- |
| Variable | CN (n= 289) | MCI (n= 164) | AD (n = 48) |
| Age (years) | 67.4 ± 6.6 | 69.5 ± 6.1 | 71.6 ± 6.3 |
| Female (%) | 154 (53%) | 91 (55%) | 27 (56%) |
| Education (years) | 16.7 ± 2.2 | 16.3 ± 2.5 | 16.2 ± 2.6 |
| APOEε4 carriers (%) | 41 (14%) | 58 (35%) | 28 (58%) |
| MMSE score | 28.7 ± 1.3 | 27.5 ± 1.4 | 24.4 ± 1.9 |
| Data are presented as mean ± standard deviation unless specified otherwise. Abbreviations: APOEε4= apolipoprotein E genotype (carrying at least one ε4 allele); MMSE= Mini-Mental State Evaluation; MCI= mild cognitive impairment. | | | |

| Supplementary Table2. Demographic and clinical characteristics of A4 | |
| --- | --- |
| Variable | CN (n= 293) |
| Age (years) | 71.2 ± 6.1 |
| Female (%) | 138 (47%) |
| Education (years) | 16.7 ± 2.2 |
| APOEε4 carriers (%) | 144 (49%) |
| MMSE score | 28.6 ± 1.5 |
| Data are presented as mean ± standard deviation unless specified otherwise. Abbreviations: APOEε4= apolipoprotein E genotype (carrying at least one ε4 allele); MMSE= Mini-Mental State Evaluation | |

**Supplementary Figure 1.** Genetic risk loci identified by FUMA analysis using GWAS data on tau/neurodegeneration residual

**Supplementary Figure 2.** Functional consequences of SNPs on genes. The proportion of SNPs (all SNPs in LD of Ind. sig. SNPs) which have corresponding functional annotations assigned by ANNOVAR.

| Supplementary Table3. Functional consequences of SNPs on genes. | | | | | | |
| --- | --- | --- | --- | --- | --- | --- |
| annot | ref.count | ref.prop | count | prop | enrichment | fisher.P |
| UTR3 | 233824 | 0.009322 | 10 | 0.019763 | 2.119909 | 0.030866 |
| UTR5 | 71546 | 0.002853 | 1 | 0.001976 | 0.692821 | 1 |
| downstream | 284177 | 0.01133 | 4 | 0.007905 | 0.697714 | 0.671592 |
| exonic | 254736 | 0.010156 | 3 | 0.005929 | 0.583764 | 0.502352 |
| intergenic | 11684523 | 0.465859 | 119 | 0.235178 | 0.504827 | 8.81E-27 |
| intronic | 9137749 | 0.36432 | 322 | 0.636364 | 1.746719 | 2.99E-35 |
| ncRNA_exonic | 259951 | 0.010364 | 6 | 0.011858 | 1.144105 | 0.65795 |
| ncRNA_intronic | 2884355 | 0.114998 | 38 | 0.075099 | 0.653042 | 0.004154 |
| ncRNA_splicing | 1313 | 5.23E-05 | 0 | 0 | 0 | 1 |
| splicing | 2830 | 0.000113 | 0 | 0 | 0 | 1 |
| upstream | 266686 | 0.010633 | 3 | 0.005929 | 0.557606 | 0.38906 |

**Supplementary Figure 3.** Enrichment of input genes in Gene Sets

**Supplementary Figure 4.** Spatial distribution of AKAP9 gene in Allen brain atlas

**Supplementary Figure 5.** Spatial distribution of STXBP6 gene in Allen brain atlas

1.	Fischl B, Salat DH, van der Kouwe AJ, Makris N, Ségonne F, Quinn BT, et al. Sequence-independent segmentation of magnetic resonance images. Neuroimage. 2004;23 Suppl 1:S69-84.
